# Cognitive differences associated with HIV serostatus and antiretroviral therapy use in a population-based sample of older adults in South Africa

**DOI:** 10.1038/s41598-020-73689-7

**Published:** 2020-10-06

**Authors:** Stephen B. Asiimwe, Meagan Farrell, Lindsay C. Kobayashi, Jen Manne-Goehler, Kathleen Kahn, Stephen M. Tollman, Chodziwadziwa Whiteson Kabudula, F. Xavier Gómez-Olivé, Ryan G. Wagner, Livia Montana, Lisa F. Berkman, M. Maria Glymour, Till Bärnighausen

**Affiliations:** 1 Department of Epidemiology and Biostatistics, University of California San Francisco, 550 16th St., Mission Hall, 2nd Floor, San Francisco, CA USA; 2 Havard Center for Population and Development Studies, Harvard University, Cambridge, MA USA; 3 Department of Epidemiology, School of Public Health, University of Michigan, Ann Arbor, MI USA; 4 Division of Infectious Diseases, Brigham and Women’s Hospital, Harvard Medical School, Boston, MA USA; 5 Africa Health Research Institute (AHRI), KwaZulu-Natal, South Africa; 6 INDEPTH Network, Accra, Ghana; 7 MRC/Wits Rural Public Health and Health Transitions Research Unit (Agincourt), School of Public Health, Faculty of Health Sciences, University of the Witwatersrand, Johannesburg, South Africa; 8 Heidelberg Institute of Global Health (HIGH), Faculty of Medicine and University Hospital, University of Heidelberg, Baden-Württemberg, Germany

**Keywords:** HIV infections, Health care, Epidemiology, Outcomes research, Translational research

## Abstract

Previous clinical studies have reported adverse cognitive outcomes for people living with HIV (PLWH), but there are no population-based studies comparing cognitive function between older PLWH and comparators without HIV in sub-Saharan Africa. We analyzed baseline data of 40 + years-old participants in “Health and Aging in Africa: A Longitudinal Study of an INDEPTH Community in South Africa” (HAALSI) cohort. We measured cognition using a battery of conventional instruments assessing orientation, immediate- and delayed-recall, and numeracy (N = 4560), and the Oxford Cognitive Screen [OCS]-Plus, a novel instrument for low-literacy populations, assessing memory, language, visual-spatial ability, and executive functioning (N = 1997). Linear regression models comparing cognitive scores between participants with and without HIV were adjusted for sex, education, age, country of birth, father’s occupation, ever-consumed alcohol, and asset index. PLWH scored on average 0.06 (95% CI 0.01–0.12) standard deviation (SD) units higher on the conventional cognitive function measure and 0.02 (95% CI − 0.07 to 0.04) SD units lower on the OCS-Plus measure than HIV-negative participants. We found higher cognitive function scores for PLWH compared to people without HIV when using a conventional measure of cognitive function but not when using a novel instrument for low-literacy settings.

## Introduction

Despite the now widespread use of antiretroviral therapy (ART) among people living with HIV (PLWH), HIV-associated neurocognitive disorder (HAND) remains a prevalent HIV-related complication^1^. HAND is sub-classified according to its progression as asymptomatic neurocognitive impairment (ANI), mild neurocognitive disorder (MND), or HIV-associated dementia (HAD)^2^. In the US, estimates of cumulative incidence of MND in ART-treated PLWH approximately one year after ART initiation ranged from 16 to 21%, and were higher than estimates of similar neurocognitive disorder in HIV-negative individuals^3,4^. Previous clinical studies in South Africa reported high prevalence of HAND among PLWH, ranging from 53^5^ to 67%^6^. However, there is no prior population-based study comparing neurocognitive impairment among PLWH (ART users and non-users) and comparable adults without HIV in sub-Saharan Africa (SSA)^7^.

The high burden of HAND in the ART era suggests that interventions over and above ART may be required to address the problem. ART is now available in the public health sector for most of SSA^8,9^, and its effects on cognition in HIV-affected populations ought to be investigated directly. Studies from high-income countries suggest persistence of cognitive impairment in the ART era at rates comparable to those in the pre-ART era. However, some studies reported change in presentation from predominantly motor skills, cognitive speed, and verbal fluency impairment in the pre-ART era, to predominantly memory and executive function impairment in the ART era, suggesting possible domain-specific effects from HIV and/or ART^10^. Even if ART does not influence cognition overall, identifying domain-specific effects of HIV and/or ART on cognition may advance our understanding of the mechanisms of cognitive impairment, potentially guiding clinical care and future research.

The context may also modify the effects of HIV and ART on cognition. For example, in SSA, PLWH often begin ART with severe levels of immunosuppression^11^. Such biological characteristics – as well as a wide range of socio-structural characteristics (e.g., amount of social-support from other family members and/or relatives who can provide care and assistance to older individuals in SSA) – could lead to different distributions of HAND in the SSA setting compared to other contexts, and possibly different impacts from ART^12^.

Measurement of cognitive function among older adults is also challenging in SSA. Conventional cognitive performance tests developed for high-literacy settings, may not be appropriate in low-literacy settings^13,14^. Previous work in the “Health and Aging in Africa: A Longitudinal Study of an INDEPTH Community in South Africa” (HAALSI) has thus developed a novel tablet-based cognitive performance instrument, the Oxford Cognitive Screen (OCS-Plus), designed for low-literacy, low-income settings. Unlike most conventional tests, which rely on reading and numeracy skills, the OCS-Plus instrument relies more on visual abilities. Literacy or numeracy skills are not required to complete the tests^15,16^.

We used HAALSI baseline data to describe in a cross-sectional design the population distribution of cognitive outcomes in a community of older PLWH residing in SSA using a battery of conventional instruments harmonized across Health and Retirement Study (HRS) sites and the novel OCS-Plus measure. Our specific aims were to: (1) compare the cognitive performance of PLWH and people without HIV; (2) among PLWH, compare cognitive performance between ART users and non-users, and (3) evaluate the performance of specific cognitive assessment instruments. We hypothesized that: (1) PLWH would have lower overall cognitive scores due to negative effects of HIV on cognitive performance; (2) among PLWH, ART users would have higher scores due to beneficial effects of ART on cognitive performance; (3) the associations of HIV and ART with cognition would vary between the conventional and the novel OCS-Plus measures; and (4) the associations of HIV and ART with cognitive scores would also vary across different domains of the OCS-Plus measure.

## Methods

### Sample

**Figure 1:**
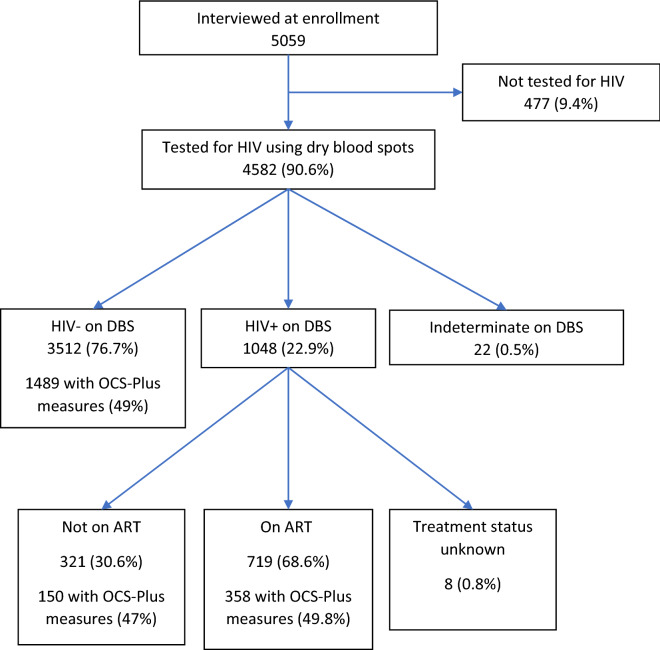
Selection of analytic sample from participants in “Health and Aging in Africa: A Longitudinal Study of an INDEPTH Community in South Africa” (HAALSI).

Participants were part of the HAALSI cohort, a population-based study designed to measure the health and well-being of adults 40 years and older in the rural Agincourt sub-district, Mpumalanga province, northeast South Africa^16^. HAALSI participants were sampled from the 2013 census round of the existing MRC/Wits-Agincourt Health and socio-Demographic Surveillance System (HDSS), which is part of the International Network for the Demographic Evaluation of Populations and their Health (INDEPTH)^17^. HAALSI data are collected using standardized tools to improve reliability. The HAALSI study enrolled 5059 men and women who were 40 years or older on July 1, 2014^17–19^. For this analysis, we restricted our sample to 4582 participants who provided dried blood spots and consented to HIV tests. We excluded participants with indeterminate HIV test results (N = 22) and HIV-positive participants whose treatment status could not be established (N = 8). We analyzed 4,560 individuals (90% of the original 5,059 HAALSI participants) (Fig. 1).

Given the prevalence of low-literacy in the sampled population, about half of HAALSI participants were randomly selected to participate in a novel cognitive function assessment, the OCS-Plus, which was designed for low-literacy and low-income settings^15^. Sampling for completion of this assessment was stratified by age and sex to allow appropriate representation.

### Predictor measurement

HIV-infection was assessed using PCR tests on dried blood spots (DBS) (Vironostika Uniform 11, Biometrica, France). Viral load measurements (Biomeriux NucliSens, Durham, NC, USA) were performed on those testing HIV-positive. DBS samples for those testing HIV-positive were also tested for emtricitabine (FTC) and lamivudine (3TC). PLWH typically receive either FTC or 3TC but not both of these commonly used antiretroviral drugs^16,18^. We regarded all HIV-positive individuals who were either virally suppressed or positive on any antiretroviral drug as ART users. A total of 662 were positive for ART and were all considered ART users (428 suppressed and 234 unsuppressed with respect to their viral load measurements). PLWH who were virally suppressed despite no evidence of being on ART (N = 52) were also classified as ART users since we did not believe that such individuals would be suppressed for reasons other than ART^20^.

### Outcome measurement

The primary outcome was the cognitive function score on a conventional measure. A previously constructed score assessing orientation, memory, and numeracy was used^21^. The battery of conventional instruments used to measure the primary outcome was intended to be harmonized across all Health and Retirement Study (HRS) sites, and included 3 measures of cognitive function: orientation, episodic memory (immediate and delayed recall) and numeracy (counting and number series)^22,23^. The tool has been validated across HRS sister sites in the US, China, and Mexico^24^. The items we used to assess orientation are also used on the standard Mini-Mental State Exam (MMSE)^25^ and the Telephone Interview for Cognitive Status (TICS)^26^. Orientation was thus assessed as the ability to state the current year, month, date, and name the current South African president (4 points). Episodic memory was assessed as immediate and delayed recall of a list of 10 words read out loud by the interviewer in xiTsonga, the local language (20 points). The delay involved 4–5 min of unrelated questions. Numeracy was assessed as the ability to count from 1 to 20 (1 point). Those able to count were further asked to complete the fourth digit of the sequence 2, 4, 6, … (1 point). Scores were combined into a z-standardized latent variable via confirmatory factor analysis in Mplus^21,27^.

Nearly half of the participants in this older-age community-representative sample were illiterate. We thus explored, in a randomly selected smaller sample, the novel Oxford Cognitive Screen (OCS)-Plus, intended to be more appropriate than conventional batteries in low-literacy, low-income settings^15^. This measure was assessed as a secondary outcome. The OCS-Plus instrument was administered in a sub-sample of HAALSI participants who had also participated in HAALSI baseline interviews. Approximately half of HAALSI baseline participants (N = 2498) were randomly selected to participate in the more extensive OCS-Plus tests. However, only those participants with known HIV and ART status were included in the present OCS-Plus analyses (N = 1997).

We used 10 items from the OCS-Plus measures, designed to tap into distinct cognitive domains and minimize education- and literacy-related bias. The OCS-Plus instrument was developed and validated in this setting by previous studies^15^. Previous factor analyses of the OCS-Plus data in HAALSI suggested 4 underlying factors: memory, language/semantic knowledge, visuospatial ability, and executive function^28^.

The memory domain included a 5-word immediate recall task, delayed word recall (4–5 min delay), and delayed word recognition, which tested ability to recognize words not retrieved during free recall (3 items, 5 points each).

The language domain was measured by a picture naming task with 4 low-frequency target pictures; and a test of semantic knowledge (4 objects), requiring participants to point to a particular object or identify the object from a specific semantic category (2 items, 4 points each).

The visuospatial domain included two tests of constructional praxis. In one test, the respondent was asked to use a stylus to copy a complex object presented on the tablet screen (figure copy). In another test, the object was briefly presented on the screen (2 s), and the respondent was asked to draw it from memory (figure recall). In each test, the object had 7 defining elements, each scored for presence (1 point), accuracy (1 point) and position (1 point) (2 items, 21 points each).

The executive function domain consisted of three different versions of a non-verbal trails task, where respondents were asked to connect shapes on the tablet screen using different rules (a point for each correct connection, 7 points maximum): first circles small to large, then squares large to small, and then alternating between circles and squares, decreasing in size for squares and increasing in size for circles (3 items, 7 points each).

### Covariate measures

**Figure 2:**
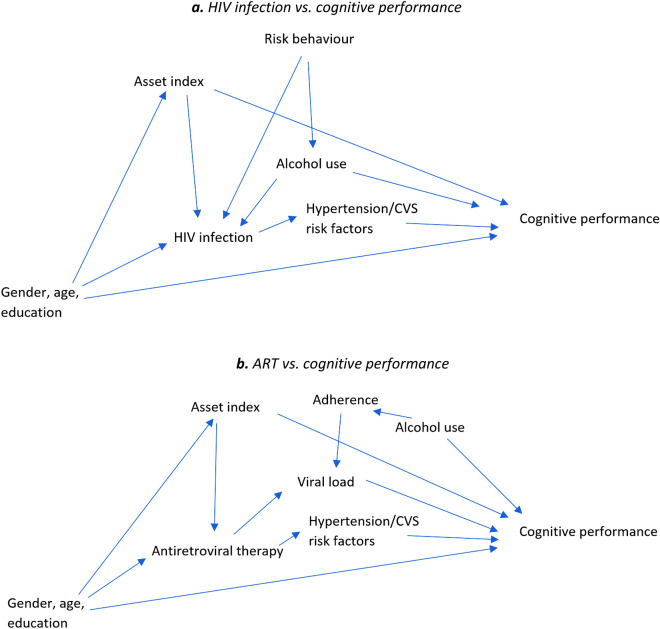
Directed acyclic graphs depicting possible relationships between HIV (**a**) and ART (**b**) and cognitive performance. (**a**) HIV infection vs. cognitive performance. (**b**) ART vs. cognitive performance.

To guide the selection of covariates into our regression models, we represented our assumptions on the underlying causal structure using a directed acyclic graph (Fig. 2). Covariate data collected during in-person interviews included father’s main occupation (manual labor vs. service sector worker vs. self-employed or business owner vs. professional vs. others), country of birth (South Africa vs. Mozambique or other), sex (male vs. female), age (years), education (no formal education vs. some primary education vs. some secondary education vs. secondary or more), ever consumed alcohol (yes vs. no), and asset index score (z-standardized score representing asset ownership, household quality and energy sources)^29^. As cardiovascular risk factors such as hypertension could also mediate effects of HIV and ART on cognition^18^, we measured systolic blood pressure (SBP) and diastolic blood pressure (DBP). Three measurements were taken two minutes apart with an electronic BP machine and the necessary cuff depending on the size of the participant (Omron, Kyoto, Japan). The average from the last two measurements was used^16,18^.

### Analyses

#### Descriptive analyses

We assessed the distributions of scores on each cognitive instrument comparing PLWH to HIV-negative individuals, and in PLWH comparing participants currently using ART to those not using ART.

#### The associations of HIV and ART use with cognitive function using conventional measures

We used linear regression to predict scores on the conventional cognitive function battery with HIV status as the predictor. We repeated this analysis using ART as the predictor, restricting to PLWH. We controlled for two sets of covariates: *model 1* included age, sex, country of birth, father’s occupation, education, asset index, and alcohol consumption; *model 2* additionally included SBP, DBP, and – when evaluating effects of ART – log-transformed viral load.

#### Derivation of domain-specific measures from the OCS-Plus

**Figure 3:**
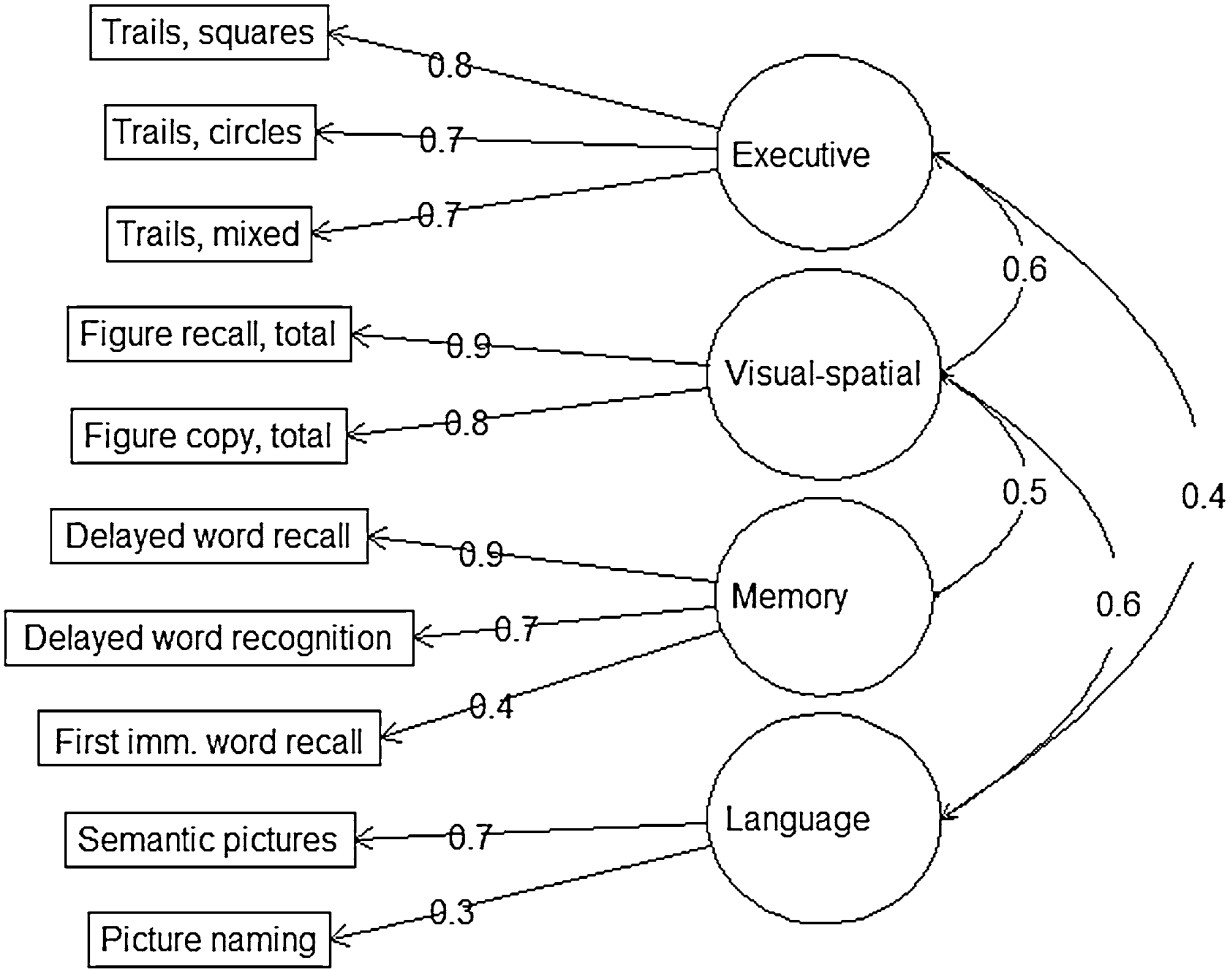
Factor loadings from a confirmatory factor analysis of 10 items from the OCS-Plus measure of cognitive function in “Health and Aging in Africa: A Longitudinal Study of an INDEPTH Community in South Africa” (HAALSI).

We used R’s Lattice package to estimate and extract z-standardized factor scores representing the four cognitive function domains of memory, language, visuospatial ability, and executive functioning from the ten individual OCS-Plus cognitive test items (Fig. 3)^30^.

#### Cognitive domain-specific effects of HIV and ART use

As a random sample of participants had undergone testing for cognition on multiple domains using the novel OCS-Plus instrument, we used this opportunity to evaluate domain-specific effects of HIV and ART on cognition, while also accounting for the possible correlation of outcomes within each individual. We thus estimated a multi-level model (Eq. 1) treating domain-specific z-scores on OCS-Plus instruments as distinct measures of cognitive function clustered within individuals. The model nested multiple cognitive assessments within individuals predicting each cognitive z-score outcome with indicator variables for each cognitive domain (memory, language, visuospatial ability, and executive functioning) and an interaction between these domains and the primary predictor (HIV or ART). Numeric covariates were centered at their means.

Specifically, using the ART use exposure model as the example and letting Y_ij_ represent cognitive z-score of individual i on domain j, we estimated multilevel models such as:

1 $${\text{Y}}_{{{\text{ij}}}} = \, \beta_{00} + \sum \beta_{{1}} {\text{M}}_{{{\text{ij}}}} + \, \beta_{{2}} {\text{A}}_{{\text{i}}} + \, \beta_{{3}} {\text{C}}_{{\text{i}}} + \sum \beta_{{4}} {\text{A}}_{{\text{i}}} *{\text{M}}_{{{\text{ij}}}} + \, \mu_{{0{\text{i}}}} + \, \varepsilon_{{{\text{ij}}}}$$

In this model, β_00_ represents average score on the reference cognitive domain (which we specified as memory in all models) among ART non-users in the reference categories of categorical covariates with the mean values of numeric covariates. M_ij_ is a set of indicator variables representing the four cognitive function domains, and β_1_ represents the corresponding coefficients (deviations from mean score in the reference outcome of memory for each cognitive domain in ART non-users). Because we estimated 4 domains, there are 3 indicator variables and corresponding coefficients, which we suppress in Eq. (1). A is an indicator variable for treatment status and β_2_ is the estimated effect of ART on memory, β_3_ represents coefficients for confounders, and β_4_ represents the set of coefficients indicating the differential effect of ART on a given cognitive domain; these coefficients test the hypothesis that the effects of ART differ across cognitive domains. Error terms μ_0i_ (representing random variability between individuals) and ε_ij_ (representing random variability within individuals) were assumed to be approximately normally distributed.

#### Interaction between age and HIV in their association with cognitive score

As age is strongly associated with cognitive function, we assessed for its interaction with HIV in predicting cognitive scores on the conventional measure and a simple average of the 4 domain-specific z-standardized scores on the OCS-Plus.

#### Missing data

For the analysis using the conventional measure of cognitive function, we conducted a complete-case analysis (only 92/4582 [2%] of participants had missing scores and less than 1% of participants were missing any covariate data). Missing data in specific items on the OCS-Plus ranged from 0.8% on picture identification to 32% on figure copy. We used multiple imputation to impute missing values on the OCS-Plus variables before confirmatory factor analysis and subsequent analyses. Multiple imputation was implemented using the MICE package in R^31^. We used predictive mean matching to estimate missing values based on 5 nearest neighbors and created five copies of the dataset with missing values imputed under a missing-at-random assumption given a participant’s age, gender, education, asset index, country of origin, HIV test results, systolic blood pressure, diastolic blood pressure, alcohol ever consumption, and father’s occupation. Subsequent analyses were performed on each of the imputed datasets and results pooled into a final estimate^32,33^.

#### Statistical significance

We judged statistical significance for all regression analyses using the 95% confidence interval (CI). Coefficients from linear regression models were considered statistically significant if their 95% CI did not include zero. Where P values are reported, statistical significance was based on a P value < 0.05 on a 2-tailed test.

### Ethics statement

The main HAALSI study was approved by the University of Witwatersrand Human Research Ethics Committee (No. M141159), the Office of Human Research Administration at the Harvard T. H. Chan School of Public Health (No. 13-1608) and the Mpumalanga Provincial Research and Ethics Committee. Secondary data analysis of HAALSI data was also approved by the Committee on Human Research at UCSF (No. 18-25025, Date: 05/09/2018). The study was conducted in accordance with relevant guidelines and regulations. Participants provided written informed consent before being interviewed and before providing any blood samples.

## Results

### Sample characteristics

The HAALSI cohort study interviewed 5059 participants at baseline. The majority of participants (4582 [90.6%] provided blood samples for HIV testing and were included in this analysis. A total of 477 individuals out of the 5059 HAALSI baseline participants (9.4%) did not provide blood samples and were thus not tested for HIV. The HIV prevalence among those tested was 1048/4582 (23%). PLWH were younger on average than HIV-negative participants (55 years [SD = 10] vs. 64 years [SD = 13]), had slightly a slightly lower proportion with no formal education (41% vs. 47%), slightly lower systolic blood pressure mean = 130 (SD = 22) versus 140 (SD = 23) mmHg, and lower average score on the wealth and asset index, -0.31 (SD = 2.2) vs. 0.1 (SD = 2.4) (Table 1). Among the 1048 PLWH analyzed, 719 (69%) were ART users. The ART users were like non-ART users with respect to all covariates except they presented with a much lower viral load (mean = 5874 copies/ml, SD = 22,610, in ART users versus 52,300, SD = 250,269 among non-ART users) (Table 1).

**Table 1:** Characteristics of baseline participants in Health and Aging in Africa: a longitudinal study of an INDEPTH Community in South Africa (HAALSI), Agincourt, South Africa, 2015, N = 4560.

Characteristic	HIV-negative (N = 3512)	All HIV-positive (N = 1048)	ART users (N = 719)	Non-ART users (N = 321)
Age, (IQR)	64 (13)	55 (10)	56 (10)	55 (11)
Men (N, %)	1614 (46%)	483 (46%)	339 (47%)	140 (43%)
**Country of birth, N (%)**				
South Africa	2486 (71%)	683 (65%)	477 (66%)	202 (64%)
Mozambique/Other	1024 (29%)	363 (35%)	240 (33%)	119 (37%)
**Father’s occupation**				
Manual labor	1950 (56%)	558 (56%)	414 (58%)	169 (53%)
Services	349 (9.9%)	104 (9.9%)	78 (11%)	26 (8.1%)
Self-employed or business	111 (3.2%	34 (3.2%	22 (3.1%)	12 (3.7%)
Professional	302 (8.6%)	106 (10%)	73 (10%)	33 (10%)
Other	387 (11%)	130 (12%)	83 (14%)	46 (14%)
Don’t know or refused	412 (12%)	85 (8.1%)	48 (11%)	35 (11%)
**Education (N, %)**				
No formal education	1660 (47%)	429 (41%)	286 (40%)	141 (44%)
Some primary education	1225 (35%)	364 (35%)	263 (37%)	96 (30%)
Some secondary education	345 (9.8%)	162 (16%)	107 (15%)	54 (17%)
Secondary education or more	272 (7.7%)	89 (8.5%)	60 (9.0%)	29 (8.3%)
Ever consumed alcohol	1564 (46%)	470 (45%)	339 (47%)	128 (40%)
Asset index score, mean (SD)	0.10 (2.4)	− 0.31 (2.2)	− 0.22 (2.2)	− 0.48 (2.2)
Systolic BP, mean (SD), mmHg	140 (23)	130 (22)	129 (22)	133 (22)
Diastolic BP, mean (SD), mmHg	83 (13)	81 (13)	80 (13)	83 (12)
Hemoglobin, mean (SD), mg/dl	12.7 (2.0)	12.1 (1.9)	12.1 (2)	12.2 (1.7)
Viral load, mean (SD), copies/ml	–	16,191 (136,351)	1499 (11,668)	49,034 (242,627)

### Distributions of cognitive function measures

PLWH had higher mean scores on the conventional cognitive function battery than HIV-negative participants. The OCS-Plus scores were similar between groups except for trail tests, where PLWH had slightly higher scores (Table 2).

**Table 2:** Cognitive outcomes on conventional and OCS-Plus cognitive batteries, according to HIV status, HAALSI, Agincourt, South Africa, 2015.

Measures	HIV negative	HIV-positive		
Conventional measures	(N = 3512)	All (N = 1048)	ART users (N = 719)	ART non-users (N = 321)
**Orientation (N giving correct response)**				
Current date, N (%)	2382 (88%)	838 (80%)	579 (81%)	252 (79%)
Current month, N (%)	2749 (78%)	916 (87%)	633 (88%)	277 (86%)
Current year, N (%)	2506 (71%)	850 (81%)	583 (81%)	262 (82%)
Current president, N (%)	2794 (80%)	893 (85%)	619 (86%)	268 (84%)
**Word recall (number of words)**				
Immediate, median (IQR)	4 (3–5)	5 (3–6)	4 (3–6)	5 (3–6)
Delayed, median (IQR)	4 (2–5)	4 (3–5)	4 (3–5)	4 (3–5)
**Numeracy (N responding correctly)**				
Forward count (1–20), N (%)	2636 (75%)	867 (83%)	605 (84%)	256 (80%)
Skip pattern, N (%)	1892 (54%)	655 (63%)	452 (63%)	198 (62%)
Cognitive score, mean (SD)	− 0.06 (0.99)	0.19 (0.94)	0.20 (0.92)	0.18 (0.99)

Measures	HIV negative	HIV-positive		
OCS-plus, correct responses, median (IQR)	N = 1489	N = 505	N = 358	N = 150
**Executive function**				
Trails, circles	3 (1–6)	4 (2–7)	4 (2–7)	4 (1–6)
Trails, squares	3 (0–5)	4 (1–5)	3 (1–5)	4 (1–5)
Trails, mixed	3 (1–6)	4 (1–7)	4 (2–7)	4 (1–6)
**Language**				
Picture naming	3 (3–4)	3 (2–3)	3 (2–3)	3 (2–3)
Semantic pictures	3 (2–4)	3 (3–4)	4 (3–4)	3 (3–4)
**Visual-spatial ability**				
Figure copy	19 (17–20)	19 (16–20)	19 (16–20)	20 (17–21)
Figure recall	17 (12–19)	17 (12–19)	16 (12–19)	18 (14–20)
**Memory**				
First immediate word recall	3 (3–4)	4 (2–4)	4 (3–4)	3 (3–4)
Delayed word recall	3 (2–4)	3 (2–4)	3 (2–4)	3 (2–4)
Delayed word recognition	5 (4–5)	5 (4–5)	5 (4–5)	5 (4–5)

### Association of HIV status and ART treatment with cognitive scores on the conventional measure

After adjustment for confounders, PLWH had 0.06 standard deviation (SD) units higher cognitive scores than HIV-negative participants on the conventional cognitive battery (β = 0.06; 95% CI 0.01–0.12). Additional adjustment for systolic blood pressure and diastolic blood pressure did not significantly alter the results (Table 3). For context, in this sample a one-year increase in age was associated with a cognitive score difference of − 0.021 (95% CI − 0.018 to − 0.023) SD units.

**Table 3:** Associations between HIV status, ART use, and the conventional cognitive battery z-score, HAALSI, Agincourt, South Africa, 2015.

	Model 1	Model 2
**HIV status**		
HIV-negative	Ref.	Ref.
HIV-positive (β, 95% CI)	0.06 (0.01 to 0.12)	0.07 (0.01 to 0.12)
**Treatment status among HIV-positive**		
ART non-user	Ref.	Ref.
ART user (β, 95% CI)	− 0.01 (− 0.12 to 0.09)	− 0.05 (− 0.20 to 0.10)

Model 1 was adjusted for age, sex, education, father’s occupation, country of birth, asset index score, ever consumed alcohol. Model 2 was adjusted for all the model 1 confounders plus systolic blood pressure, diastolic blood pressure, and in analyses of the effects of ART use status, viral load.

Among PLWH, we did not find evidence for significant differences in cognitive score by ART use status. In a sensitivity analysis comparing PLWH using ART and PLWH not using ART to HIV negative individuals, there were no significant differences in cognitive scores between ART users and non-users, although both groups had higher scores on the conventional measure than HIV-negative individuals (Table 4).

**Table 4:** Association of HIV status by ART use status with cognitive scores using a three-level categorical variable of HIV-positive ART users vs. HIV-positive ART non-users vs. HIV-negative participants with HIV-negative participants as reference.

	Model 1	Model 2
**Conventional measure**		
HIV-negative	Ref.	Ref.
HIV-positive ART user	0.06 (− 0.001 to 0.13)	0.07 (0.002 to 0.13)
HIV-positive ART non-user	0.06 (− 0.03 to 0.15)	0.07 (− 0.03 to 0.16)
**Summary OCS-plus measure**		
HIV-negative	Ref.	Ref.
HIV-positive ART user	− 0.04 (− 0.10 to 0.03)	− 0.03 (− 0.09 to 0.04)
HIV-positive ART non-user	− 0.07 (− 0.17 to 0.03)	− 0.07 (− 0.17 to 0.03)

### Association of HIV infection with cognitive function on the OCS-plus instrument

In the multi-level model adjusting for covariates, there were no significant domain-specific interactions for the effects of HIV on cognition (Table 5). In a model to estimate the overall effect of HIV and ART on cognition using the OCS Plus instrument (the main effects model, dropping the measure by exposure interaction), PLWH had 0.02 SD units lower cognitive scores than HIV-negative participants, although the result was not statistically significant (β = − 0.02; 95% CI − 0.07 to 0.04). Among PLWH, ART users had higher cognitive scores than non-ART users (overall ART effects from a main-effects model), but the confidence interval was wide and included the null (β = 0.04; 95% CI − 0.07 to 0.14) (Table 5). Similar findings were observed in additional models, where the outcome was a simple average of the scores on the 4 OCS-Plus domains (Table 6).

**Table 5:** Associations between HIV status, ART use, and domain-specific cognitive function as measured on the OCS-Plus instrument, HAALSI, Agincourt, South Africa, 2015.

	Main effect		Domain specific effect							
			Memory		Language		Visual-spatial		Executive	
	Model 1	Model 2	Model 1	Model 2	Model 1	Model 2	Model 1	Model 2	Model 1	Model 2
**HIV status**										
Negative	Ref.	Ref.	Ref.	Ref.	Ref.	Ref.	Ref.	Ref.	Ref.	Ref.
Positive	− 0.02 (− 0.07 to 0.04)	− 0.01 (− 0.07 to 0.05)	− 0.003 (− 0.07 to 0.08)	− 0.006 (− 0.07 to 0.08)	− 0.05 (− 0.13 to 0.04)	− 0.05 (− 0.13 to 0.04)	− 0.03 (− 0.11 to 0.06)	− 0.03 (− 0.11 to 0.06)	− 0.01 (− 0.09 to 0.08)	− 0.01 (− 0.09 to 0.08)
**ART status**										
Non-user	Ref.	Ref.	Ref.	Ref.	Ref.	Ref.	Ref.	Ref.	Ref.	Ref.
ART user	0.04 (− 0.07 to 0.14)	0.01 (− 0.15 to 0.14)	0.08 (− 0.06 to 0.22)	0.04 (− 0.14 to 0.22)	− 0.04 (− 0.19 to 0.12)	− 0.04 (− 0.20 to 0.12)	− 0.12 (− 0.28 to − 0.04)	− 0.12 (− 0.28 to − 0.04)	− 0.02 (− 0.18 to 0.13)	− 0.02 (− 0.18 to 0.13)

The domain specific effect was estimated from a model with an interaction term between the measure and the predictor. The main effect is estimated without the interaction term between measure and predictor. Model 1 was adjusted for age, sex, education, country of origin, father’s occupation, asset index score, and alcohol ever use. Model 2 was further adjusted for blood pressure, and in those with HIV for the ART use analysis, viral load.

**Table 6:** Association of HIV status and treatment status with a summary OCS-Plus measure of cognition.

	Model 1	Model 2
**HIV status**		
HIV-negative	Ref.	Ref.
HIV-positive (β, 95% CI)	− 0.03 (− 0.10 to 0.03)	− 0.03 (− 0.09 to 0.04)
**Treatment status among HIV-positive**		
ART non-user	Ref.	Ref.
ART user (β, 95% CI)	0.03 (− 0.09 to 0.15)	0.01 (− 0.17 to 0.19)

### Interaction between age and HIV in their association with cognitive score

**Figure 4:**
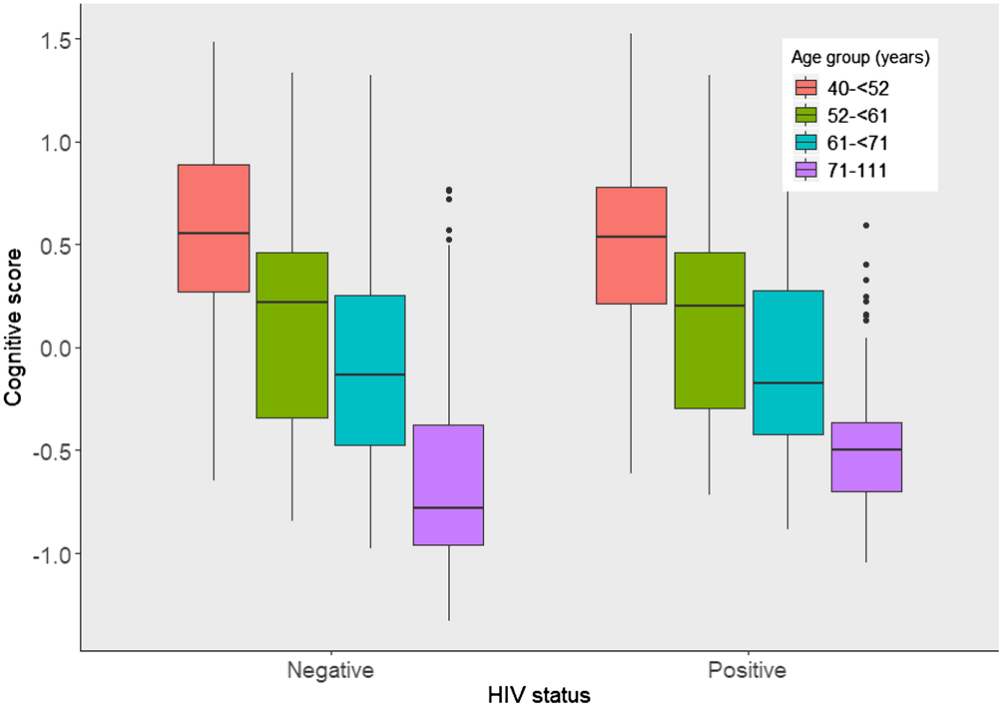
Age-HIV interaction in predicting cognitive score on the conventional measure by quartiles of age.

**Figure 5:**
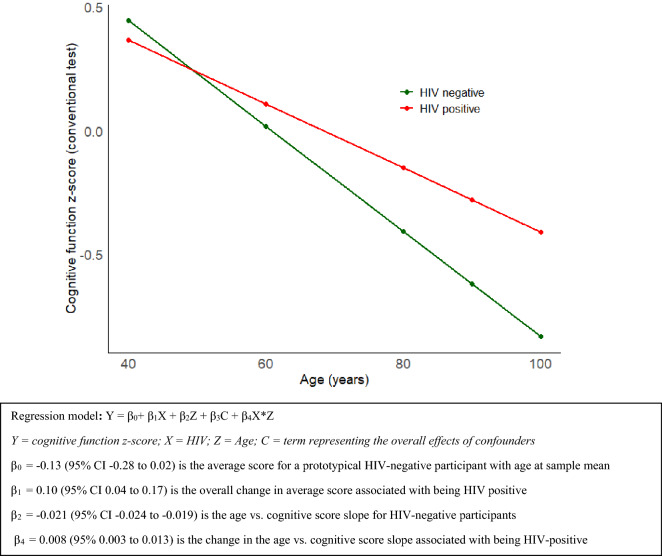
Age-HIV interaction in predicting cognitive score on the conventional measure with age as a continuous variable.

In the analysis using the conventional cognitive function measure, older PLWH had higher cognitive scores than older participants without HIV (Figs. 4 and 5). In a model to predict scores on the conventional cognitive function measure, adjusting for covariates, and including an interaction term between centered age and HIV status, PLWH had 0.11 SD units higher overall cognitive scores (β = 0.11, 95% CI 0.05–0.18). In a similar model using the average of scores on the 4 OCS-Plus domains as the outcome, PLWH averaged 0.078 SD units lower scores on (β = – 0.078, 95% CI – 0.14 to 0.001), though the result was not statistically significant, and there was no interaction between age and HIV status (β = − 0.01, 95% CI − 0.01 to 0.001) (Table 7).

**Table 7:** Interaction between age and HIV in their association with cognition.

	Conventional measure	OCS-plus measure
HIV-negative	Ref.	Ref.
HIV-positive (β, 95% CI)	0.10 (0.04–0.17)	− 0.078 (− 0.14 to 0.001)
Centered age (β, 95% CI)	− 0.021 (− 0.024 to − 0.019)	− 0.014 (− 0.017 to − 0.010)
HIV-positive: age (β, 95% CI)	0.008 (0.003–0.013)	− 0.01 (− 0.01 to 0.001)

### Sensitivity analyses

We performed a sensitivity analysis to assess whether the exclusion of some of the HAALSI baseline participants, who were not tested for HIV, may have substantially influenced our findings. We found only a few significant differences between those who were tested for HIV and those not tested for HIV. For example, there was no major difference between those tested and those not tested on age, education, country of origin, father’s occupation, hemoglobin and blood pressure (Supplementary Table S1). Those not tested had a higher proportion of men (51%) compared to those tested (46%, P = 0.04) and higher scores on the asset index (mean = 0.25, versus 0.005 for those who were tested, P = 0.03).

Among the 4582 HAALSI baseline participants who were tested for HIV and who were thus analyzed for this report, sex was associated with cognitive score (men had higher scores, P < 0.01) but not HIV (P = 0.94). The asset index was associated with HIV; compared to people without HIV, PLWH had lower average scores on the asset index. The asset index was also associated with cognitive function; participants with higher scores on the asset index also had higher cognitive scores on the conventional measure and the OCS plus measure.

Additional sensitivity analyses conducted among PLWH further assessed the impact of treatment status and viral suppression status on cognitive scores. We did not see significant differences between the cognitive scores of individuals who were suppressed despite no evidence of being on ART versus other PLWH. We also did not see any differences in cognitive score between those who were suppressed on ART versus those who were unsuppressed on ART or unsuppressed and not on ART (Supplementary Table S2).

## Discussion

This study presents, for the first time to our knowledge, a comparison of the cognitive function according to HIV and ART use status in older people in a rural region of sub-Saharan Africa. The comparison was done in groups with similar sociodemographic characteristics, in an HIV hyperendemic community in rural South Africa. When cognitive function was measured by a conventional battery commonly used in high-income countries, we observed an average of 0.06 SD units higher cognitive function scores among PLWH, equivalent to a 3-year cognitive advantage for PLWH. However, when cognitive function was measured using the OCS-Plus, which uses visual and auditory cues to minimize literacy-related bias in cognitive testing in low-literacy communities, we did not observe any significant differences in cognitive function according to HIV and ART use status^15^. When using this measure, PLWH had an average of 0.03 SD units lower overall cognitive function scores than people without HIV. This association was not statistically significant, but, within our data, it was equivalent to a 1.5 year cognitive disadvantage for PLWH. ART use was non-significantly associated with better cognitive outcomes among PLWH.

Our results represent a unique comparison of people living with HIV (PLWH) and comparable older adults without HIV in a rural setting in sub-Saharan Africa in the context of wide ART availability through the public health sector. Our observations on the conventional measure were different from previous observations in high-income settings whereby PLWH tend to have lower cognitive scores than people without HIV. In a US study, older PLWH had slightly higher cumulative incidence of MND (5% vs. 3%) compared to HIV-negative participants^4^. For SSA, we did not find similar population-based studies, but in a hospital-based study of individuals with psychosis, PLWH were more likely to be cognitively impaired^34^. Our findings using the OCS-Plus are more consistent with the expectation that PLWH would have worse cognitive function than comparators without HIV.

Our observations on the conventional measure, whereby PLWH have better cognition than persons without HIV were similar to the findings of previous studies in SSA which investigated non-cognition outcomes. Findings of better health for PLWH in comparisons with people without HIV are not unprecedented in this setting. Some population-based studies of diabetes and hypertension in the same setting have reported better outcomes for PLWH. In a previous study using HAALSI data, PLWH averaged lower SBP and lower prevalence of diabetes^18^. In a Ugandan study, HIV infection was associated with approximately 3.3 mmHg lower SBP and 30% lower odds of hypertension^35^. These observations were from different outcomes but suggest that PLWH in this setting may have better health outcomes than people without HIV, and this may be related to increased use of ART among PLWH^36,37^.

Differential access to healthcare for PLWH compared to people without HIV is a possible explanation of a health advantage among PLWH. In a previous study using data from HAALSI, PLWH were more likely to have had their blood pressure and blood sugar measured, more likely to have received counseling on exercise, more likely to be aware of their hypertension status and, as such, more likely to have been treated^18^. In HAALSI, a large proportion (69%) of PLWH is receiving ART. HIV care programs in SSA are often vertical programs that are more intensively managed, which may lead to better services than the general healthcare system serving individuals without HIV^38,39^. It is unclear if these mechanisms might contribute to better cognitive outcomes for PLWH, and this may warrant future study.

Selective survival among PLWH may also explain apparent cognitive advantage, if more vulnerable PLWH died before cross-sectional sampling of the HAALSI baseline data occurred. A selected group of PLWH who are robust to the physical and cognitive consequences of HIV may then be sampled. This mechanism is consistent with our analyses assessing for interaction between age and HIV on the conventional measure of cognitive function where older PLWH had higher cognitive scores than HIV-negative comparators. Whereas our regression model results show that overall, PLWH have higher scores than people without HIV, the results from our sensitivity analyses, focusing on age strata, show that the group primarily driving this difference is individuals aged 70 + . In younger age strata, PLWH and people without HIV have relatively similar cognition. These observations may indeed support the possibility of selective survival. Future longitudinal studies are important to better understand this issue.

Other selection processes influencing initial infection risk, sampling, or data availability may also shape cognitive patterns in the HAALSI sample. For example, very old and very sick PLWH might not have provided blood samples for HIV testing. Cognitive advantage however was not observed among PLWH on the OCS-Plus measure, raising doubts on this mechanism. Numerous unmeasured factors may also have influenced HIV infection and cognition since, despite adjustment for confounders, we cannot rule out the possibility of residual confounding in an observational study. In particular, literacy bias could give advantage to PLWH if literacy differences are not adequately controlled for by adjustment for education level. Unlike the conventional measure, the OCS-Plus measure relies more on auditory and visual cues and may not have been affected by literacy bias in our analysis. There was also a significant age difference between PLWH (relatively younger) and persons without HIV (relatively older). However, we do not expect these age differences to explain our results since age was adjusted for in our regression models. Sensitivity analyses accounting for any non-linear age effects yielded similar findings.

Our findings using the OCS-Plus were inconclusive, but they were more consistent with our prior expectation of a cognitive disadvantage among PLWH. OCS-plus captures multiple domains of cognitive function; some of these domains may be particularly sensitive to HIV associated brain changes. Even though we did not see domain-specific differences here, our findings overall suggest that different measures can produce different conclusions, and that the sensitivity, validity, and cultural relevance of cognitive measures are of critical importance when examining the relationship between HIV and cognitive function. Future studies using the OCS-Plus and other novel cognitive function assessment tools designed to be more appropriate in low-literacy settings are recommended^40^. Such novel tools when validated in population-based studies could then be adopted for use in clinical settings to assess cognitive function.

Our results should not be interpreted as causal given the several competing alternative explanations. Regardless, even if not causal, our findings have important implications for policies aimed at providing care to aging cohorts of PLWH in SSA. If PLWH are systematically more likely to have cognitive impairment, policies to provide for HIV treatment and management would need to be designed to accommodate such impairments. We do not find evidence in our data that such policies are needed at this time, suggesting that current guidelines, whereby screening for cognitive impairments in asymptomatic PLWH is generally not recommended, should be sustained^41^. Although selective survival within PLWH is a possible explanation for our findings, ART treatment occurring early in the course of HIV-infection for most PLWH, which is likely to happen with the current policy of “test and treat”, may mitigate such selective survival and enable better future studies of the effects of both HIV and ART on cognition.

A cognitive advantage in PLWH, if confirmed may mean that a growing number of older adults living with HIV may not necessarily cause an epidemic of HIV-associated dementia. A broader range of interventions and programs to provide for ongoing management of HIV infection in older adults may then be needed. But even then, support to those with more severe cognitive impairment and/or their caregivers may be needed, since social programs to support elderly and cognitively impaired individuals are largely unavailable in SSA. In our study, we only detected a non-significant beneficial effect from ART on cognitive score. We did not observe any difference by viral suppression status irrespective of ART use status. Our inability to establish duration of treatment, and the possibility that people not on ART at the time of data collection (before the adoption of the current “test and treat” policy for HIV infection) may have been generally healthier than people on ART, are both limitations to our observations. Future studies that can better evaluate ART effects on cognition are thus suggested.

Our study provides unique data comparing HIV infected individuals to uninfected individuals with similar sociodemographic backgrounds in a setting of high HIV prevalence and where ART is now widely available through the public health sector. Objective measures on HIV status and ART use, as well as multiple cognitive function measures add to the strengths of our observations. However, out data are also limited by their cross-sectional nature; our findings do not necessarily describe causal effects of HIV on cognition. We were unable to analyze some participants since they did not provide samples for HIV testing. In a sensitivity analysis, those not tested were more likely to be men and had higher asset index scores. We suspect that the slight influence of sex on testing is unlikely to account for our results. The asset index was associated with HIV and with cognitive score. Our sample may thus have been enriched for people of lower SES who are also likely to have HIV and more likely to have low cognitive function scores. However, in our results, PLWH had higher scores on the conventional measure. We therefore suspect that the exclusion of some participants is unlikely to be biasing the relationship between HIV and cognition in the analytic sample.

We recommend future longitudinal studies to further explore cognitive differences between HIV-positive and HIV-negative individuals in this setting and to further develop measurement tools that may be more appropriate for this and similar settings. Such longitudinal studies will help us to determine whether the superior cognitive function of PLWH that we observed on the conventional cognitive function measure represents a real biopsychosocial phenomenon or was driven by selection or confounding biases that cannot be disentangled with cross-sectional data.

In conclusion, in this population-based study of older individuals living in an HIV hyperendemic community of sub-Saharan Africa, PLWH had higher cognitive function scores than comparable people without HIV on a conventional cognitive function assessment but not on a novel measure designed for low-literacy settings. Possible explanations include differential participation in testing, selective survival, cognitive function recovery with ART, and residual confounding. However, the observation may also suggest that PLWH could have better clinical outcomes than people without HIV. Although the observed difference is modest, it is non-trivial and equates to a 3-year age advantage on cognition for PLWH. Findings on the novel OCS-Plus measure were more consistent with prior expectation. Future studies to further validate novel tools for the assessment of cognitive function in this setting are suggested. Once validated in population-based studies, these tools could be extended to the measurement of cognitive function in clinical practice.

## Supplementary information

Supplementary file 1.

## Data Availability

Most HAALSI data are available to the public on this website: https://dataverse.harvard.edu/. Biomarker data are not publicly available but can be provided to interested parties on request. Interested parties can contact the study team by email (HAALSI@hsph.harvard.edu) for access to biomarker data.

## Abbreviations

ANI: Asymptomatic neurocognitive impairment
ART: Antiretroviral therapy
DBP: Diastolic blood pressure
DBS: Dried blood spots
FTC: Emtricitabine
HAALSI: Health and aging in Africa: a longitudinal study of an INDEPTH community in South Africa
HAD: HIV-associated dementia
HAND: HIV-associated neurocognitive disorder
HDSS: Health and socio-demographic surveillance system
INDEPTH: International network for the demographic evaluation of populations and their health
MICE: Multiple imputation by chained equations
MRC: Medical research council
OCS-Plus: Oxford cognitive screen-plus
PLWH: People living with HIV
PCR: Polymerase chain reaction
SBP: Systolic blood pressure
SSA: Sub-Saharan Africa
3TC: Lamivudine
