# Supplementary material for: Cognitive differences associated with HIV serostatus and antiretroviral therapy use in a population-based sample of older adults in South Africa
Source: Sci Rep. 2020 Oct 6;10:16625. doi: 10.1038/s41598-020-73689-7 (PMC7539005; doi:10.1038/s41598-020-73689-7)
Supplement: Supplementary file 1 [file 41598_2020_73689_MOESM1_ESM.docx]

**Cognitive differences associated with HIV serostatus and antiretroviral therapy use in a population-based sample of older adults in South Africa**

Stephen B. Asiimwe, MD, PhD^1^, Meagan Farrell, PhD^2^, Lindsay C. Kobayashi, PhD^3^, Jen Manne-Goehler, MD^4^, Kathleen Kahn, MD, PhD^5,6^, Stephen M. Tollman, MMed, PhD^5,6^, Chodziwadziwa Whiteson Kabudula, PhD^6^, F. Xavier Gómez-Olivé, PhD^6^, Ryan G. Wagner, PhD^6^, Livia Montana, PhD^2^, Lisa F. Berkman, PhD^2,6^, M. Maria Glymour, ScD^1^, and Till Bärnighausen, MD, ScD, PhD^2,4,6,7^

^1^University of California San Francisco, California, USA

^2^Havard Center for Population and Development Studies, Harvard University, Massachusetts, USA

^3^Department of Epidemiology, School of Public Health, University of Michigan, Ann Arbor, Michigan, USA

^4^Africa Health Research Institute (AHRI), KwaZulu-Natal, South Africa

^5^INDEPTH Network, Accra, Ghana

^6^MRC/Wits Rural Public Health and Health Transitions Research Unit (Agincourt), School of Public Health, Faculty of Health Sciences, University of the Witwatersrand, Johannesburg, South Africa.

^7^Heidelberg Institute of Global Health (HIGH), Faculty of Medicine and University Hospital, University of Heidelberg, Baden-Württemberg, Germany

**Funding**

The HAALSI study is funded by the National Institute on Aging (NIA) of the National Institutes of Health (NIH) (P01-AG41710 and 5P01AG041710-05). The Agincourt Health Surveillance System site is supported by the University of Witwatersrand, Medical Research Council, South Africa, and the Wellcome Trust, UK (058893/Z/99/A; 069683/Z/02/Z; 085477/Z/08/Z; 085477/B/08/Z). TB was supported by the Alexander von Humboldt Foundation through the Alexander von Humboldt Professor award, funded by the Federal Ministry of Education and Research; the Welcome Trust; and from the National Institute of Child and Human Development (NICHD) of NIH (R01-HD084233), National Institute of Allergy and Infectious Diseases (NIAID) of NIH (R01-AI124389 and R01-AI112339) as well as FIC of NIH (D43-TW009775).

**Conflicts of interest and source of funding**: No conflict of interest declared.

**Correspondence**

Stephen B. Asiimwe, Department of Epidemiology and Biostatistics, University of California San Francisco, 550 16^th^ St, Mission Hall, 2^nd^ Floor, San Francisco, CA. Email: [asiimwesteve@gmail.com](mailto:asiimwesteve@gmail.com)

**Supplementary table 1**. The characteristics of HAALSI participants who were tested for HIV versus those not tested at cohort entry (2014).

|  | **Tested for HIV (N = 4582)** | **Not tested for HIV (N = 477)** |
| --- | --- | --- |
| **Age, mean (SD)** | 62 (13) | 62 (15) |
| **Men (%)**^*^ | 46% | 51% |
| **Education (%)** |  |  |
| No education | 46% | 44% |
| Some primary education | 35% | 25% |
| Some secondary education | 11 % | 14% |
| Secondary education or more | 8% | 17% |
| **Father’s occupation (%)** |  |  |
| Manual labor | 56% | 54% |
| Services | 10% | 10% |
| Self-employed or business | 3% | 3% |
| Professional | 9% | 9% |
| Other | 11% | 12% |
| Don’t know or refused | 11% | 12% |
| **Country of origin (%)** |  |  |
| South Africa | 70% | 72% |
| Mozambique or other | 30% | 28% |
| **Asset index score, mean (SD)**^*^ | 0.005 (2.36) | 0.25 (2.42) |
| **Ever consumed alcohol (%)** | 45% | 44% |
| **Hemoglobin, mean (SD)** | 13.0 (2.4) | 12.4 (2.3) |
| **Systolic blood pressure, mean (SD)** | 138 (25) | 138 (23) |
| **Diastolic blood pressure, mean (SD)** | 82 (13) | 82 (14) |

^*^Significantly different between those tested and those not tested for HIV

**Supplementary table 2**. Treatment status and suppression status and relationship with cognitive scores. Linear regression coefficients from models including treatment and suppression status dummies alongside covariates. Model 1 is adjusted for sociodemographic covariates. Model 2 is further adjusted for blood pressure.

| *Conventional measure* | *Model 1* | *Model 2* |
| --- | --- | --- |
| Suppressed on ART (N = 428) | Ref. | Ref. |
| Suppressed but no evidence of ART use (52) | -0.12 (-0.35 to 0.11) | -0.12 (-0.35 to 0.11) |
| Unsuppressed on ART (234) | -0.04 (-0.17 to 0.09) | -0.05 (-0.18 to 0.07) |
| Unsuppressed and not on ART (N = 320) | -0.005 (-0.12 to 0.11) | -0.03 (-0.15 to 0.09) |
| *OCS Plus measure* |  |  |
| Suppressed on ART | Ref. | Ref. |
| Suppressed but no evidence of ART use | 0.07 (-0.14 to 0.28) | 0.08 (-0.13 to 0.29) |
| Unsuppressed on ART | -0.01 (-0.14 to 0.12) | -0.01 (-0.14 to 0.11) |
| Unsuppressed and not on ART | -0.05 (-0.17 to 0.07) | -0.06 (-0.18 to 0.06) |

**Health and Aging in Africa: A Longitudinal Study of an INDEPTH Community in South Africa (HAALSI)**

**Conventional cognitive function measures**

*Domains: Memory; Numeracy; Orientation*

| **Memory domain** |  |  |
| --- | --- | --- |
| **Field name** | **Instructions and question** | **Response option and data value** |
| CN004 | We are going to read a list consisting of 10 words and we would like you to memorize as many as you can. We deliberately made the list long to make it difficult for anyone to memorize all of the words; most people will only remember a few of them. Please listen carefully as we read the list because we cannot repeat it. When we finish reading the list, we will ask you to recall and tell us as many words as you can remember, and they don’t have to be in the order that you heard them. Is this explanation clear? [IWER: IF NO: EXPLAIN] | YES................................................1  NO ................................................2 |
| CN005 | Randomized variable: [WHICH WORD LIST] |  |
| CN006 | [IWER: READ THE LIST SLOWLY, WITH AN INTERVAL OF ABOUT 2 SECONDS BETWEEN EACH WORD. DO NOT ALLOW PROXY ANSWERS.]  Rice  River  Doctor  Clothes  Egg  Cat  Bowl  Child  Hand  Book |  |
|  |  |  |
| CN007 | Now please let us know the words you are able to recall. [IWER: INSTRUCTIONS: GIVE RESPONDENT ENOUGH TIME TO RECALL, APPROXIMATELY UP TO 2 MINUTES.] [IWER: INSTRUCTIONS: CIRCLE ALL THE WORDS MENTIONED BY THE RESPONDENT ON THE COLUMN.] | Rice…………………………………………..1 River………………………………………….2 Doctor……………………………………….3 Clothes………………………………………4 Egg…………………………………………….5Cat…………………………………………….6 Bowl………………………………………….7 Child………………………………………….8 Hand………………………………………….9 Book………………………………………..10  None recalled………………………….88 |
|  |  |  |
| CN008 | [IWER: INSTRUCTIONS: READ THE SAME LIST ONCE MORE, UP TO 3 TIMES, AND THEN GO ON. IF RESPONDENT DOES NOT RECALL ANY OF THE WORDS, ASSURE THEM THAT IT IS OK SO THAT RESPONDENT WILL FEEL COMFORTABLE/AT EASE.]  Rice  River  Doctor  Clothes  Egg  Cat  Bowl  Child  Hand  Book |  |
| CN009 | Try to remember the words I just read to you. I'll ask you to recall them later.  Please count from 1 to 20 | Counted correctly 1 to 20…………..1 Cannot count 1-20……………………..2 Counted incorrectly 1-20……………3 |
| **Numeracy domain** |  |  |
| CN010 | [IWER: MAKE SURE RESPONDENT HAS PENCIL AND PAPER READY FOR WRITING DOWN THE NUMBERS. REPEAT INSTRUCTIONS FOR THE RESPONDENT IF NECESSARY. ALLOW ENOUGH TIME WHEN READING NUMBERS FOR RESPONDENT TO WRITE DOWN SEQUENCE.]  Next, I'm going to read you several numbers and I'd like you to write them down from left to right. There will be a blank number in the series that I read to you. Draw a dash or short blank line when I say “blank.” Then look at the pattern of numbers. Based on this pattern, tell me what number goes in the blank. Sometimes the blank will be at the end of the series, and sometimes the blank will be in the beginning or in the middle. For example, if I said the numbers ‘1. . . 2. . . BLANK. . .4’ then what number would go in the blank?  [IWER: THE CORRECT RESPONSE IS 3. IF RESPONDENT DOES NOT GIVE THE CORRECT RESPONSE “3” THEN SAY: “THE ANSWER WE WERE LOOKING FOR IS 3.” PROBE IF NEEDED TO CHECK THAT THE RESPONDENT UNDERSTANDS THE TASK, BY ASKING: “DO YOU UNDERSTAND THE DIRECTIONS FOR THIS TASK?”] |  |
| CN011 | I’m going to read you a series of numbers. There will be a blank number in the series that I read to you. I would like you to write down the numbers from left to right and then tell me what number goes in the blank based on the pattern of numbers. 2. . . 4. . . 6. . .BLANK [IWER: NOW PLEASE LOOK AT THE NUMBER YOU JUST WROTE DOWN AND TELL ME THE NUMBER THAT GOES IN THE BLANK.] [IWER: THE SEQUENCE IS 2 4 6 8. 8 IS THE ANSWER WE WERE LOOKING FOR BECAUSE, IN THIS EXAMPLE, THE NUMBERS INCREASE BY 2.] | 8…………………………………………………1 Any number other than 8…………..2 Does not know answer………………3 Respondent does not understand instructions………………………………..4 |
| **Memory domain** |  |  |
| CN012 | A little while ago, I read you a list of words and you repeated the ones you could remember. Please tell me any of the words that you remember now. [IWER: ANSWERS ARE DISPLAYED ONLY FOR INTERVIEWER. PLEASE DO NOT SHOW THE SCREEN TO RESPONDENT.] [IWER: CHECK ALL THE WORDS MENTIONED BY THE RESPONDENT AND LIST ALL OF THE WORDS MENTIONED WHICH ARE NOT ON THE LIST] | Rice…………………………………………..1 River………………………………………….2 Doctor……………………………………….3 Clothes………………………………………4 Egg…………………………………………….5Cat…………………………………………….6 Bowl………………………………………….7 Child………………………………………….8 Hand………………………………………….9 Book………………………………………..10  Not on the list………………………….11  Not on the list………………………….12  Not on the list………………………….13  Not on the list………………………….14  Not on the list………………………….15  None recalled………………………….16 |
|  |  |  |
| CN013 | [IWER: HOW OFTEN DID THE RESPONDENT RECEIVE ASSISTANCE IN ANSWERING THIS SECTION?] | Never………………………………………..1  A few times……………………………….2  Most or all of the time……………….3 |
| **Orientation domain** |  |  |
| CN014 | What is the year we are in now? | Correct……………………………………….1  Incorrect…………………………………….2 |
|  |  |  |
| CN015 | Can you tell me the month? | Correct……………………………………….1  Incorrect…………………………………….2 |
|  |  |  |
| CN016 | What is the date today? (what day of the month is it today) | Correct……………………………………….1  Incorrect…………………………………….2 |
|  |  |  |
| CN017 | Who is the current president? | Correct……………………………………….1  Incorrect…………………………………….2 |

**Reference**

HAALSI data website: https://dataverse.harvard.edu/dataset.xhtml?persistentId=doi:10.7910/DVN/F5YHML

**Health and Aging in Africa: A Longitudinal Study of an INDEPTH Community in South Africa (HAALSI)**

**Detailed Description of the Oxford Cognitive Screen-Plus.**

The Oxford Cognitive Screen-Plus (OCS-Plus) for the HAALSI tests (**Humphreys et al., 2017; Farrell et al., 2020**) is run as an offline application on Windows Surface Pro tablets. A dedicated stylus is used to record responses controlled by the interviewer or the respondent depending on the task. Standard administration and scoring procedures are ensured using interviewer scripts and automated scoring. The domains outlined below were included in HAALSI tests:

***Picture Naming.*** Respondents are asked to name four grayscale line drawings of concrete objects. The drawings depict objects with low-to-medium frequency names, and interviewers are instructed to count as correct only the pre-defined object names. One point is assigned for each correct answer, for a total score ranging from 0 to 4.

***Semantics.*** Four sets of known objects are presented. For the first two sets, respondents are asked to point to specific objects (i.e., point to the pineapple), and for the second two sets are asked to point to an object from a semantic category (i.e., point to the bird). One point is assigned for each correct answer, for a total score ranging from 0 to 4.

***Orientation.*** To evaluate awareness of time and place, respondents are asked open-ended questions about the year, month, date and president. One point is assigned for each correct answer, for a total score ranging from 0 to 4.

***Immediate Word Recall/Word Encoding****.* The interviewer reads a list of 5 words to the respondent, who is immediately asked to recall as many of the words on the list as possible (first immediate recall). The interviewer reads the same list to the respondent who is asked to recall them for a second time (second immediate recall). The outcome measure for this task is the sum of immediate recall trials, for a total score ranging from 0 to 10.

***Trails.*** An array of dark gray circles and light gray squares of different sizes is presented on the tablet screen, and respondents use the stylus to draw a line connecting the objects in a specific order, the rules for which change across trials. The task consists of two baseline test conditions and one set-switching test condition. Interviewer demonstration and two practice runs are conducted prior to each test trial. For baseline 1, respondents are instructed to connect circles from small to large (ignoring squares). For baseline 2, respondents are instructed to connect squares from large to small (ignoring circles). For the set-switching trial, respondents are asked to alternate between circles and squares, decreasing size for squares and increasing size for circles. Respondents are given a point for each correct connection, for a maximum score of 7 on each baseline trial and 14 on the set-switching trial.

***Word Recall and Word Recognition***. After an average 4 to 5 minute delay, respondents are asked to recall as many words as possible from the previously learned 5-item word list (delayed recall). For any word not recalled, respondents were presented with a multiple-choice list of four words, each list consisting of the target word and three semantically-related distractors (delayed word recognition). One point is assigned for each word correctly recalled or recognized, for a total score of 0-5 for the delayed recall task and 0-5 for the recognition task. Respondents who recall all words on the recall task are assigned the maximum score on recognition.

***Incidental Memory.*** The incidental memory task evaluates the respondents’ memory for stimuli that they are not told to remember. Respondents are presented three sets of four line drawings and asked to indicate which item they have previously seen. Each set of four items contains only one picture presented earlier in the testing session: a target item from the picture-naming task and two distractor items from the semantics task. A fourth trial tests for word memory, with one target from the word recall list and three distractors. One point is assigned for each recognized item, for a total score ranging from 0 to 4.

***Rule Finding.*** This task requires respondents to predict the movement of a red dot as it moves through an array of squares and triangles arranged in a grid. The dot moves in a lawful manner but frequently switches its operating rule, requiring respondents to adaptively learn the new rule. Rule changes are not signaled, and respondents are not given explicit feedback about the correctness of their responses. Prior to each move, respondents use the stylus to indicate the position where they think the dot would move to next. A point is awarded for each rule learned, defined as correctly predicting two consecutive moves within a rule. The task consists of five rules, for a total score ranging from 0 to 5.

***Figure Copy and Figure Recall.*** During the figure copy portion, a complex object is presented on the top of the screen and the respondent instructed to copy the figure into the space below it using the stylus. For figure recall, the same complex object is presented at the top of the screen and removed after 2 seconds. The respondent is asked to draw the object from memory. The objects produced during both portions of the task are scored by two trained raters. The complex object consists of seven elements, and each element is assigned a score for presence (0-1), accuracy of the image (0-1), and position (0-1). Total scores for both the figure copy and the figure recall tasks range from 0 to 21.

***Auditory Attention.*** Selective attention is evaluated by presenting blocks of audio-recorded words and instructing participants to make different responses depending on whether the word is a target or nontarget word. There are three target words (goodbye, down, no), three semantically related distractors (hello, up, yes), and three unrelated distractors (and, eat, day). Each target is presented nine times over three blocks, with randomization of word order and interstimulus interval (1, 2, or 3 seconds). Accuracy scores for target trials (0-27) and combined distractor trials were derived (0-54).

**References**

Farrell, M.T., Kobayashi, L.C., Montana, L., Wagner, R.G., Demeyere, N., Berkman, L.F., 2020. **Education disparity partially explains cognitive gender differences in older rural South Africans**. *The Journals of Gerontology, Series B: Psychological Sciences and Social Sciences (Online ahead of print).*

Humphreys GW, Duta MD, Montana L, et al. **Cognitive Function in Low-Income and Low-Literacy Settings: Validation of the Tablet-Based Oxford Cognitive Screen in the Health and Aging in Africa: A Longitudinal Study of an INDEPTH Community in South Africa (HAALSI)**. *J Gerontol B Psychol Sci Soc Sci.* 2017;72(1):38-50.
